# Bis(4-ethyl­benzoato-κ*O*)bis­(nicotin­amide-κ*N* ^1^)zinc(II)

**DOI:** 10.1107/S1600536811006830

**Published:** 2011-02-26

**Authors:** Hacali Necefoğlu, Füreya Elif Özbek, Vedat Aktaş, Barış Tercan, Tuncer Hökelek

**Affiliations:** a Department of Chemistry, Kafkas University, 36100 Kars, Turkey; b Department of Physics, Karabük University, 78050, Karabük, Turkey; c Department of Physics, Hacettepe University, 06800 Beytepe, Ankara, Turkey

## Abstract

The title Zn^II^ complex, [Zn(C_9_H_9_O_2_)_2_(C_6_H_6_N_2_O)_2_], contains two 4-ethyl­benzoate and two nicotinamide monodentate ligands, leading to a distorted tetrahedral coordination of the Zn^II^ ion. The dihedral angles between the carboxyl­ate groups and the adjacent benzene rings are 10.33 (13) and 2.38 (11)°, while opposite pyridine and benzene rings are oriented at dihedral angles of 68.46 (5) and 81.09 (6)°. In the crystal, inter­molecular N—H⋯O hydrogen bonds link the mol­ecules, forming a three-dimensional network. C—H⋯O inter­actions also occur as well as two weak C—H⋯π inter­actions involving the benzene rings.

## Related literature

For niacin, see: Krishnamachari (1974 ▶). For the nicotinic acid derivative *N*,*N*-diethyl­nicotinamide, see: Bigoli *et al.* (1972 ▶). For related structures, see: Hökelek *et al.* (1996 ▶, 2009*a* ▶,*b* ▶); Hökelek & Necefoğlu (1998 ▶, 2007 ▶). For standard bond lengths, see: Allen *et al.* (1987 ▶).
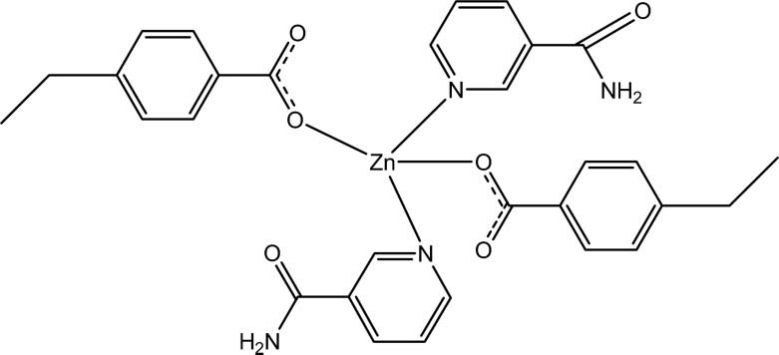


## Experimental

### 

#### Crystal data

[Zn(C_9_H_9_O_2_)_2_(C_6_H_6_N_2_O)_2_]
*M* *_r_* = 607.97
Monoclinic, 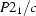

*a* = 8.0601 (2) Å
*b* = 15.9736 (3) Å
*c* = 21.2568 (3) Å
β = 94.384 (3)°
*V* = 2728.78 (9) Å^3^
*Z* = 4
Mo *K*α radiation
μ = 0.95 mm^−1^
*T* = 100 K
0.31 × 0.30 × 0.27 mm

#### Data collection

Bruker Kappa APEXII CCD area-detector diffractometer
Absorption correction: multi-scan (*SADABS*; Bruker, 2001 ▶) *T*_min_ = 0.752, *T*_max_ = 0.763
26561 measured reflections
6812 independent reflections
5761 reflections with *I* > 2σ(*I*)
*R*_int_ = 0.045

#### Refinement

*R*[*F* ^2^ > 2σ(*F* ^2^)] = 0.032
*wR*(*F* ^2^) = 0.078
*S* = 1.03
6812 reflections
388 parameters
H atoms treated by a mixture of independent and constrained refinement
Δρ_max_ = 0.33 e Å^−3^
Δρ_min_ = −0.44 e Å^−3^

### 

Data collection: *APEX2* (Bruker, 2007 ▶); cell refinement: *SAINT* (Bruker, 2007 ▶); data reduction: *SAINT*; program(s) used to solve structure: *SHELXS97* (Sheldrick, 2008 ▶); program(s) used to refine structure: *SHELXL97* (Sheldrick, 2008 ▶); molecular graphics: *ORTEP-3 for Windows* (Farrugia, 1997 ▶); software used to prepare material for publication: *WinGX* (Farrugia, 1999 ▶) and *PLATON* (Spek, 2009 ▶).

## Supplementary Material

Crystal structure: contains datablocks I, global. DOI: 10.1107/S1600536811006830/su2256sup1.cif

Structure factors: contains datablocks I. DOI: 10.1107/S1600536811006830/su2256Isup2.hkl

Additional supplementary materials:  crystallographic information; 3D view; checkCIF report

## Figures and Tables

**Table 1:** Hydrogen-bond geometry (Å, °)

*D*—H⋯*A*	*D*—H	H⋯*A*	*D*⋯*A*	*D*—H⋯*A*
N3—H32⋯O4^i^	0.86 (2)	1.99 (2)	2.833 (2)	165 (2)
N4—H41⋯O5^ii^	0.89 (2)	2.07 (2)	2.947 (2)	169 (2)
N4—H42⋯O6^iii^	0.84 (2)	2.06 (2)	2.901 (2)	177 (2)
C6—H6⋯O2^ii^	0.95	2.59	3.412 (2)	145
C19—H19⋯O5^ii^	0.95	2.29	3.2277 (19)	168
C21—H21⋯O2^i^	0.95	2.58	3.497 (2)	161
C23—H23⋯O3	0.95	2.49	3.085 (2)	121
C29—H29⋯O5^ii^	0.95	2.45	3.299 (2)	149
C17—H17*A*⋯*Cg*1^iv^	0.99	2.63	3.436 (2)	139
C20—H20⋯*Cg*1^v^	0.95	2.77	3.603 (2)	147

Symmetry codes: (i) 
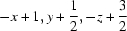
; (ii) 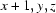; (iii) 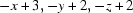; (iv) 
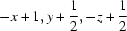
; (v) 
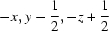
.

**Fig. 1.**
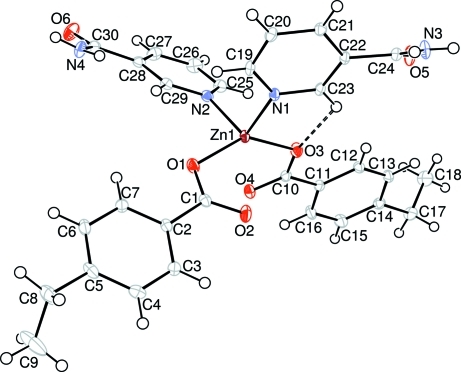
The molecular structure of the title molecule with the atom-numbering scheme. Displacement ellipsoids are drawn at the 50% probability level. The intramolecular C-H···O hydrogen bond is shown as a dashed line.

**Fig. 2.**
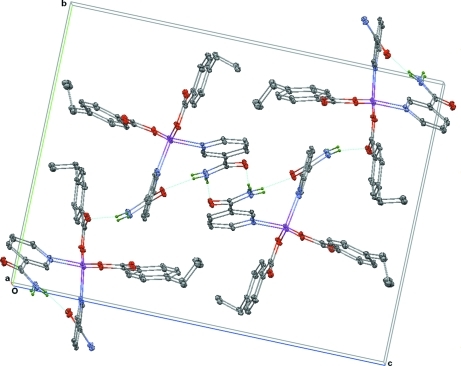
A view along the a-axis of the crystal packing of the title compound. The N-H···O hydrogen bonds are shown as dashed cyan lines [H-atoms not involved in hydrogen bonding have been omitted for clarity].

## supplementary crystallographic information

### Comment

As a part of our ongoing investigations of transition metal complexes of nicotinamide (NA), one form of niacin (Krishnamachari, 1974), and/or the nicotinic acid derivative *N*,*N*-diethylnicotinamide (DENA), an important respiratory stimulant (Bigoli *et al.*, 1972), the title compound was synthesized and its crystal structure is on reported herein.

The title complex, (Fig. 1), is a mononuclear Zn^II^complex, consisting of two nicotinamide (NA) and two 4-ethylbenzoate (PEB) ligands, all ligandscoordinating in a monodentate manner. The crystal structures of similar complexes of Cu^II^, Co^II^, Ni^II^, Mn^II^ and Zn^II^ ions, [Cu(C_7_H_5_O_2_)_2_(C_10_H_14_N_2_O)_2_] (Hökelek *et al.*, 1996), [Co(C_6_H_6_N_2_O)_2_(C_7_H_4_NO_4_)_2_(H_2_O)_2_] (Hökelek & Necefoğlu, 1998), [Ni(C_7_H_4_ClO_2_)_2_(C_6_H_6_N_2_O)_2_(H_2_O)_2_] (Hökelek *et al.*, 2009*a*), [Mn(C_9_H_10_NO_2_)_2_(H_2_O)_4_]2H_2_O] (Hökelek & Necefoğlu, 2007) and [Zn(C_7_H_4_BrO_2_)_2_(C_6_H_6_N_2_O)_2_(H_2_O)_2_] (Hökelek *et al.*, 2009*b*) have also been reported. In the copper(II) complex mentioned above the two benzoate ions coordinate to the Cu^II^ atom as bidentate ligands, while in the other structures all the ligands coordinate in a monodentate manner.

In the title complex the near equality of the C1—O1 [1.282 (2) Å], C1—O2 [1.238 (2) Å] and C10—O3 [1.283 (2) Å], C1—O2 [1.243 (2) Å] bonds in the carboxylate groups indicate delocalized bonding arrangements, rather than localized single and double bonds. The Zn—O bond lengths are 1.9321 (12) and 1.9470 (11) Å, and the Zn—N bond lengths are 2.0525 (13) and 2.0767 (14) Å, close to standard values (Allen *et al.*, 1987). The Zn atom is displaced out of the least-squares planes of the carboxylate groups (O1/C1/O2) and (O3/C10/O4) by -0.1444 (2) and -0.1364 (2) Å, respectively. The dihedral angles between the planar carboxylate groups and the adjacent benzene rings A (C2—C7) and B (C11—C16) are 10.33 (13) and 2.38 (11) °, respectively. The benzene A (C2—C7) and B (C11—C16) rings and the pyridine C (N1/C19—C23) and D (N2/C25—C29) rings are oriented at dihedral angles of A/B = 81.09 (6), A/C = 80.79 (5), A/D = 31.68 (5), B/C = 12.68 (5), B/D = 70.61 (6) and C/D = 68.46 (5) °.

In the crystal intermolecular N—H···O link the molecules to form a three-dimensional network (Table 1 and Fig. 2). There also C-H···O interactions, and two weak C—H···π interactions involving the benzene ring A (C2-C7) (Table 1).

Footnote for Table 1: Cg1 is the centroid of ring A (C2-C7.)

### Experimental

The title compound was prepared by the reaction of ZnSO_4_.H_2_O (0.89 g, 5 mmol) in H_2_O (100 ml) and NA (1.22 g, 10 mmol) in H_2_O (50 ml) with sodium 4-ethylbenzoate (1.72 g, 10 mmol) in H_2_O (100 ml) at room temperature. The mixture was filtered and set aside to crystallize at ambient temperature for two weeks, giving colourless single crystals.

### Refinement

Atoms H31, H32, H41 and H42 (for the NH_2_ groups) were located in a difference Fourier map and were freely refined. The C-bound H-atoms were positioned geometrically with C—H = 0.95, 0.99 and 0.98 Å, for aromatic, methylene and methyl H-atoms, respectively, and constrained to ride on their parent atoms, with *U*_iso_(H) = k × *U*_eq_(C), where k = 1.5 for methyl H-atoms and k = 1.2 for all other H-atoms.

### Crystal data

**Table:**

[Zn(C_9_H_9_O_2_)_2_(C_6_H_6_N_2_O)_2_]	*F*(000) = 1264
*M**_r_* = 607.97	*D*_x_ = 1.480 Mg m^−^^3^
Monoclinic, *P*2_1_/*c*	Mo *K*α radiation, λ = 0.71073 Å
Hall symbol: -P 2ybc	Cell parameters from 9453 reflections
*a* = 8.0601 (2) Å	θ = 2.3–28.3°
*b* = 15.9736 (3) Å	µ = 0.95 mm^−^^1^
*c* = 21.2568 (3) Å	*T* = 100 K
β = 94.384 (3)°	Block, colourless
*V* = 2728.78 (9) Å^3^	0.31 × 0.30 × 0.27 mm
*Z* = 4	

### Data collection

**Table:**

Bruker Kappa APEXII CCD area-detector diffractometer	6812 independent reflections
Radiation source: fine-focus sealed tube	5761 reflections with *I* > 2σ(*I*)
graphite	*R*_int_ = 0.045
φ and ω scans	θ_max_ = 28.3°, θ_min_ = 1.9°
Absorption correction: multi-scan (*SADABS*; Bruker, 2001)	*h* = −10→10
*T*_min_ = 0.752, *T*_max_ = 0.763	*k* = −18→21
26561 measured reflections	*l* = −25→28

### Refinement

**Table:**

Refinement on *F*^2^	Primary atom site location: structure-invariant direct methods
Least-squares matrix: full	Secondary atom site location: difference Fourier map
*R*[*F*^2^ > 2σ(*F*^2^)] = 0.032	Hydrogen site location: inferred from neighbouring sites
*wR*(*F*^2^) = 0.078	H atoms treated by a mixture of independent and constrained refinement
*S* = 1.03	*w* = 1/[σ^2^(*F*_o_^2^) + (0.0293*P*)^2^ + 1.6476*P*] where *P* = (*F*_o_^2^ + 2*F*_c_^2^)/3
6812 reflections	(Δ/σ)_max_ = 0.002
388 parameters	Δρ_max_ = 0.33 e Å^−^^3^
0 restraints	Δρ_min_ = −0.44 e Å^−^^3^

### Special details

**Table:**

Geometry. All e.s.d.'s (except the e.s.d. in the dihedral angle between two l.s. planes) are estimated using the full covariance matrix. The cell e.s.d.'s are taken into account individually in the estimation of e.s.d.'s in distances, angles and torsion angles; correlations between e.s.d.'s in cell parameters are only used when they are defined by crystal symmetry. An approximate (isotropic) treatment of cell e.s.d.'s is used for estimating e.s.d.'s involving l.s. planes.
Refinement. Refinement of *F*^2^ against ALL reflections. The weighted *R*-factor *wR* and goodness of fit *S* are based on *F*^2^, conventional *R*-factors *R* are based on *F*, with *F* set to zero for negative *F*^2^. The threshold expression of *F*^2^ > σ(*F*^2^) is used only for calculating *R*-factors(gt) *etc*. and is not relevant to the choice of reflections for refinement. *R*-factors based on *F*^2^ are statistically about twice as large as those based on *F*, and *R*- factors based on ALL data will be even larger.

### Fractional atomic coordinates and isotropic or equivalent isotropic displacement parameters (Å^2^)

**Table:**

	*x*	*y*	*z*	*U*_iso_*/*U*_eq_	
Zn1	0.71491 (2)	0.888748 (11)	0.825485 (9)	0.01161 (6)	
O1	0.91423 (14)	0.87417 (7)	0.78163 (6)	0.0171 (2)	
O2	0.73308 (14)	0.85419 (8)	0.69894 (6)	0.0209 (3)	
O6	1.35846 (16)	0.92561 (8)	1.03534 (6)	0.0241 (3)	
O3	0.50224 (14)	0.83456 (7)	0.83563 (6)	0.0172 (2)	
O4	0.66060 (14)	0.72104 (8)	0.84067 (6)	0.0187 (3)	
O5	0.13462 (14)	1.06652 (8)	0.83268 (6)	0.0199 (3)	
N1	0.63168 (16)	1.00911 (8)	0.81069 (6)	0.0120 (3)	
N2	0.84101 (17)	0.88825 (8)	0.91441 (7)	0.0133 (3)	
N3	0.14554 (18)	1.16536 (9)	0.75686 (7)	0.0168 (3)	
H31	0.047 (3)	1.1708 (14)	0.7533 (11)	0.030 (6)*	
H32	0.203 (3)	1.1909 (14)	0.7303 (11)	0.028 (6)*	
N4	1.32842 (19)	0.98657 (10)	0.93949 (7)	0.0185 (3)	
H41	1.260 (3)	1.0050 (14)	0.9080 (11)	0.028 (6)*	
H42	1.421 (3)	1.0104 (14)	0.9461 (11)	0.030 (6)*	
C1	0.87746 (19)	0.86154 (10)	0.72266 (8)	0.0136 (3)	
C2	1.02295 (19)	0.85648 (10)	0.68257 (8)	0.0130 (3)	
C3	1.0005 (2)	0.83375 (10)	0.61948 (8)	0.0164 (3)	
H3	0.8932	0.8177	0.6020	0.020*	
C4	1.1337 (2)	0.83427 (11)	0.58184 (8)	0.0192 (3)	
H4	1.1173	0.8170	0.5391	0.023*	
C5	1.2923 (2)	0.86004 (10)	0.60619 (9)	0.0189 (3)	
C6	1.3143 (2)	0.88062 (10)	0.66957 (8)	0.0178 (3)	
H6	1.4216	0.8963	0.6873	0.021*	
C7	1.1815 (2)	0.87866 (10)	0.70766 (8)	0.0148 (3)	
H7	1.1992	0.8925	0.7511	0.018*	
C8	1.4347 (2)	0.86644 (12)	0.56405 (10)	0.0290 (4)	
H8A	1.4025	0.9057	0.5292	0.035*	
H8B	1.5321	0.8907	0.5888	0.035*	
C9	1.4854 (3)	0.78609 (14)	0.53652 (14)	0.0498 (7)	
H9A	1.5744	0.7963	0.5085	0.075*	
H9B	1.3897	0.7609	0.5124	0.075*	
H9C	1.5257	0.7480	0.5705	0.075*	
C10	0.52318 (19)	0.75551 (10)	0.84359 (7)	0.0137 (3)	
C11	0.37299 (19)	0.70554 (10)	0.85754 (8)	0.0134 (3)	
C12	0.22077 (19)	0.74497 (10)	0.86294 (8)	0.0141 (3)	
H12	0.2122	0.8039	0.8578	0.017*	
C13	0.0817 (2)	0.69897 (10)	0.87579 (8)	0.0158 (3)	
H13	−0.0207	0.7270	0.8802	0.019*	
C14	0.0898 (2)	0.61218 (10)	0.88240 (8)	0.0156 (3)	
C15	0.2425 (2)	0.57307 (10)	0.87673 (9)	0.0193 (3)	
H15	0.2506	0.5140	0.8810	0.023*	
C16	0.3833 (2)	0.61926 (10)	0.86493 (9)	0.0184 (3)	
H16	0.4868	0.5917	0.8619	0.022*	
C17	−0.0651 (2)	0.56207 (11)	0.89316 (9)	0.0196 (4)	
H17A	−0.0353	0.5019	0.8950	0.024*	
H17B	−0.1468	0.5701	0.8565	0.024*	
C18	−0.1475 (2)	0.58478 (12)	0.95274 (9)	0.0268 (4)	
H18A	−0.2483	0.5511	0.9553	0.040*	
H18B	−0.1770	0.6443	0.9517	0.040*	
H18C	−0.0703	0.5736	0.9897	0.040*	
C19	0.73450 (19)	1.07550 (10)	0.80977 (8)	0.0141 (3)	
H19	0.8512	1.0661	0.8127	0.017*	
C20	0.6764 (2)	1.15687 (10)	0.80474 (8)	0.0152 (3)	
H20	0.7525	1.2023	0.8050	0.018*	
C21	0.50608 (19)	1.17165 (10)	0.79933 (7)	0.0132 (3)	
H21	0.4633	1.2270	0.7952	0.016*	
C22	0.39986 (18)	1.10297 (10)	0.80012 (7)	0.0118 (3)	
C23	0.46747 (18)	1.02318 (10)	0.80640 (7)	0.0122 (3)	
H23	0.3943	0.9767	0.8077	0.015*	
C24	0.21397 (19)	1.11059 (10)	0.79769 (8)	0.0138 (3)	
C25	0.7753 (2)	0.85405 (10)	0.96462 (8)	0.0169 (3)	
H25	0.6625	0.8362	0.9606	0.020*	
C26	0.8661 (2)	0.84391 (11)	1.02190 (8)	0.0210 (4)	
H26	0.8169	0.8187	1.0564	0.025*	
C27	1.0297 (2)	0.87093 (10)	1.02831 (8)	0.0192 (3)	
H27	1.0946	0.8639	1.0672	0.023*	
C28	1.0980 (2)	0.90846 (10)	0.97729 (8)	0.0142 (3)	
C29	0.9994 (2)	0.91520 (10)	0.92118 (8)	0.0137 (3)	
H29	1.0458	0.9400	0.8859	0.016*	
C30	1.2733 (2)	0.94122 (10)	0.98604 (8)	0.0155 (3)	

### Atomic displacement parameters (Å^2^)

**Table:**

	*U*^11^	*U*^22^	*U*^33^	*U*^12^	*U*^13^	*U*^23^
Zn1	0.00833 (9)	0.01253 (10)	0.01407 (10)	0.00004 (6)	0.00148 (6)	0.00079 (7)
O1	0.0135 (5)	0.0229 (6)	0.0154 (6)	0.0017 (4)	0.0041 (4)	0.0004 (5)
O2	0.0100 (5)	0.0268 (7)	0.0258 (7)	−0.0015 (5)	0.0014 (5)	−0.0005 (5)
O6	0.0236 (7)	0.0271 (7)	0.0201 (7)	−0.0052 (5)	−0.0084 (5)	0.0055 (5)
O3	0.0125 (5)	0.0151 (6)	0.0243 (7)	−0.0024 (4)	0.0033 (5)	0.0017 (5)
O4	0.0113 (5)	0.0216 (6)	0.0237 (7)	0.0008 (5)	0.0034 (5)	−0.0034 (5)
O5	0.0098 (5)	0.0289 (7)	0.0211 (6)	−0.0008 (5)	0.0016 (5)	0.0101 (5)
N1	0.0091 (6)	0.0147 (6)	0.0121 (6)	−0.0001 (5)	0.0010 (5)	0.0004 (5)
N2	0.0139 (6)	0.0112 (6)	0.0151 (7)	0.0005 (5)	0.0024 (5)	−0.0002 (5)
N3	0.0089 (7)	0.0203 (7)	0.0214 (8)	0.0024 (5)	0.0017 (6)	0.0070 (6)
N4	0.0127 (7)	0.0249 (8)	0.0173 (8)	−0.0021 (6)	−0.0033 (6)	0.0027 (6)
C1	0.0135 (7)	0.0091 (7)	0.0185 (8)	0.0007 (6)	0.0036 (6)	0.0012 (6)
C2	0.0117 (7)	0.0115 (7)	0.0161 (8)	0.0008 (6)	0.0028 (6)	0.0012 (6)
C3	0.0137 (7)	0.0166 (8)	0.0189 (8)	0.0002 (6)	0.0005 (6)	−0.0017 (6)
C4	0.0238 (9)	0.0188 (8)	0.0154 (8)	0.0030 (7)	0.0047 (7)	−0.0017 (7)
C5	0.0189 (8)	0.0148 (8)	0.0244 (9)	0.0032 (6)	0.0102 (7)	0.0003 (7)
C6	0.0115 (7)	0.0169 (8)	0.0252 (9)	0.0003 (6)	0.0035 (6)	−0.0001 (7)
C7	0.0136 (7)	0.0149 (8)	0.0160 (8)	0.0001 (6)	0.0010 (6)	−0.0002 (6)
C8	0.0267 (10)	0.0268 (10)	0.0360 (12)	−0.0003 (8)	0.0190 (8)	−0.0021 (8)
C9	0.0552 (15)	0.0309 (12)	0.0703 (18)	0.0036 (11)	0.0491 (14)	−0.0030 (11)
C10	0.0114 (7)	0.0171 (8)	0.0125 (8)	−0.0016 (6)	0.0016 (6)	−0.0020 (6)
C11	0.0111 (7)	0.0151 (8)	0.0142 (8)	−0.0016 (6)	0.0016 (6)	−0.0016 (6)
C12	0.0132 (7)	0.0116 (7)	0.0178 (8)	0.0000 (6)	0.0021 (6)	−0.0010 (6)
C13	0.0112 (7)	0.0159 (8)	0.0205 (9)	−0.0001 (6)	0.0027 (6)	−0.0018 (6)
C14	0.0153 (8)	0.0156 (8)	0.0161 (8)	−0.0035 (6)	0.0019 (6)	−0.0010 (6)
C15	0.0204 (8)	0.0122 (8)	0.0255 (9)	0.0002 (6)	0.0033 (7)	0.0012 (7)
C16	0.0126 (7)	0.0174 (8)	0.0253 (9)	0.0032 (6)	0.0028 (6)	−0.0004 (7)
C17	0.0186 (8)	0.0163 (8)	0.0244 (9)	−0.0080 (6)	0.0042 (7)	−0.0004 (7)
C18	0.0251 (9)	0.0271 (10)	0.0292 (10)	−0.0082 (8)	0.0091 (8)	0.0000 (8)
C19	0.0078 (7)	0.0183 (8)	0.0160 (8)	−0.0011 (6)	0.0001 (6)	0.0015 (6)
C20	0.0121 (7)	0.0155 (8)	0.0180 (8)	−0.0040 (6)	0.0011 (6)	0.0003 (6)
C21	0.0126 (7)	0.0131 (7)	0.0139 (8)	0.0001 (6)	0.0003 (6)	0.0005 (6)
C22	0.0085 (7)	0.0157 (8)	0.0113 (7)	−0.0008 (6)	0.0006 (5)	0.0004 (6)
C23	0.0091 (7)	0.0147 (7)	0.0128 (8)	−0.0023 (6)	0.0006 (6)	0.0002 (6)
C24	0.0102 (7)	0.0157 (8)	0.0155 (8)	−0.0003 (6)	0.0005 (6)	−0.0018 (6)
C25	0.0184 (8)	0.0156 (8)	0.0171 (8)	−0.0011 (6)	0.0045 (6)	0.0002 (6)
C26	0.0274 (9)	0.0199 (9)	0.0163 (9)	−0.0031 (7)	0.0046 (7)	0.0033 (7)
C27	0.0246 (9)	0.0179 (8)	0.0145 (8)	0.0000 (7)	−0.0022 (7)	0.0011 (6)
C28	0.0165 (8)	0.0110 (7)	0.0150 (8)	0.0019 (6)	0.0009 (6)	−0.0012 (6)
C29	0.0151 (7)	0.0121 (7)	0.0140 (8)	0.0017 (6)	0.0030 (6)	0.0002 (6)
C30	0.0165 (8)	0.0144 (8)	0.0152 (8)	0.0020 (6)	−0.0012 (6)	−0.0029 (6)

### Geometric parameters (Å, °)

**Table:**

Zn1—O1	1.9321 (12)	C9—H9C	0.9800
Zn1—O3	1.9470 (11)	C10—C11	1.498 (2)
Zn1—N1	2.0525 (13)	C11—C12	1.392 (2)
Zn1—N2	2.0767 (14)	C11—C16	1.389 (2)
O1—C1	1.282 (2)	C12—H12	0.9500
O2—C1	1.2380 (19)	C13—C12	1.385 (2)
O6—C30	1.233 (2)	C13—H13	0.9500
O3—C10	1.283 (2)	C14—C13	1.394 (2)
O4—C10	1.2427 (19)	C14—C17	1.515 (2)
O5—C24	1.237 (2)	C15—C14	1.394 (2)
N1—C19	1.347 (2)	C15—H15	0.9500
N1—C23	1.3387 (19)	C16—C15	1.392 (2)
N2—C25	1.343 (2)	C16—H16	0.9500
N2—C29	1.344 (2)	C17—C18	1.518 (3)
N3—C24	1.323 (2)	C17—H17A	0.9900
N3—H31	0.80 (2)	C17—H17B	0.9900
N3—H32	0.86 (2)	C18—H18A	0.9800
N4—C30	1.330 (2)	C18—H18B	0.9800
N4—H41	0.88 (2)	C18—H18C	0.9800
N4—H42	0.84 (2)	C19—C20	1.383 (2)
C1—C2	1.504 (2)	C19—H19	0.9500
C2—C3	1.388 (2)	C20—C21	1.389 (2)
C2—C7	1.393 (2)	C20—H20	0.9500
C3—C4	1.387 (2)	C21—C22	1.392 (2)
C3—H3	0.9500	C21—H21	0.9500
C4—C5	1.404 (2)	C22—C23	1.389 (2)
C4—H4	0.9500	C22—C24	1.500 (2)
C5—C6	1.385 (3)	C23—H23	0.9500
C5—C8	1.512 (2)	C25—C26	1.381 (2)
C6—C7	1.391 (2)	C25—H25	0.9500
C6—H6	0.9500	C26—C27	1.385 (3)
C7—H7	0.9500	C26—H26	0.9500
C8—C9	1.481 (3)	C27—C28	1.389 (2)
C8—H8A	0.9900	C27—H27	0.9500
C8—H8B	0.9900	C28—C29	1.386 (2)
C9—H9A	0.9800	C28—C30	1.505 (2)
C9—H9B	0.9800	C29—H29	0.9500
			
O1—Zn1—O3	140.48 (5)	C13—C12—H12	119.7
O1—Zn1—N1	108.24 (5)	C12—C13—C14	121.02 (15)
O1—Zn1—N2	94.11 (5)	C12—C13—H13	119.5
O3—Zn1—N1	98.81 (5)	C14—C13—H13	119.5
O3—Zn1—N2	105.74 (5)	C13—C14—C17	120.53 (15)
N1—Zn1—N2	105.93 (5)	C15—C14—C13	118.14 (15)
C1—O1—Zn1	110.62 (10)	C15—C14—C17	121.30 (15)
C10—O3—Zn1	110.12 (10)	C14—C15—H15	119.5
C19—N1—Zn1	123.05 (10)	C16—C15—C14	121.02 (15)
C23—N1—Zn1	118.53 (10)	C16—C15—H15	119.5
C23—N1—C19	118.23 (14)	C11—C16—C15	120.25 (15)
C25—N2—Zn1	121.96 (11)	C11—C16—H16	119.9
C25—N2—C29	118.31 (14)	C15—C16—H16	119.9
C29—N2—Zn1	119.39 (11)	C14—C17—C18	114.69 (15)
C24—N3—H31	119.8 (17)	C14—C17—H17A	108.6
C24—N3—H32	121.6 (15)	C14—C17—H17B	108.6
H32—N3—H31	118 (2)	C18—C17—H17A	108.6
C30—N4—H41	121.4 (15)	C18—C17—H17B	108.6
C30—N4—H42	117.8 (16)	H17A—C17—H17B	107.6
H41—N4—H42	118 (2)	C17—C18—H18A	109.5
O1—C1—C2	115.52 (14)	C17—C18—H18B	109.5
O2—C1—O1	123.58 (15)	C17—C18—H18C	109.5
O2—C1—C2	120.90 (15)	H18A—C18—H18B	109.5
C3—C2—C1	120.81 (14)	H18A—C18—H18C	109.5
C3—C2—C7	118.99 (15)	H18B—C18—H18C	109.5
C7—C2—C1	120.13 (15)	N1—C19—C20	122.43 (14)
C2—C3—H3	119.8	N1—C19—H19	118.8
C4—C3—C2	120.44 (15)	C20—C19—H19	118.8
C4—C3—H3	119.8	C19—C20—C21	119.51 (15)
C3—C4—C5	120.81 (16)	C19—C20—H20	120.2
C3—C4—H4	119.6	C21—C20—H20	120.2
C5—C4—H4	119.6	C20—C21—C22	118.03 (15)
C4—C5—C8	120.82 (17)	C20—C21—H21	121.0
C6—C5—C4	118.24 (15)	C22—C21—H21	121.0
C6—C5—C8	120.93 (16)	C21—C22—C24	123.30 (14)
C5—C6—C7	120.96 (16)	C23—C22—C21	119.15 (14)
C5—C6—H6	119.5	C23—C22—C24	117.48 (14)
C7—C6—H6	119.5	N1—C23—C22	122.64 (14)
C2—C7—H7	119.8	N1—C23—H23	118.7
C6—C7—C2	120.48 (16)	C22—C23—H23	118.7
C6—C7—H7	119.8	O5—C24—N3	124.20 (15)
C5—C8—H8A	108.6	O5—C24—C22	119.60 (14)
C5—C8—H8B	108.6	N3—C24—C22	116.20 (14)
C9—C8—C5	114.85 (17)	N2—C25—C26	122.23 (16)
C9—C8—H8A	108.6	N2—C25—H25	118.9
C9—C8—H8B	108.6	C26—C25—H25	118.9
H8A—C8—H8B	107.5	C25—C26—C27	119.13 (16)
C8—C9—H9A	109.5	C25—C26—H26	120.4
C8—C9—H9B	109.5	C27—C26—H26	120.4
C8—C9—H9C	109.5	C26—C27—C28	119.27 (16)
H9A—C9—H9B	109.5	C26—C27—H27	120.4
H9A—C9—H9C	109.5	C28—C27—H27	120.4
H9B—C9—H9C	109.5	C27—C28—C30	118.54 (15)
O3—C10—C11	116.75 (14)	C29—C28—C27	118.02 (15)
O4—C10—O3	122.55 (15)	C29—C28—C30	123.43 (15)
O4—C10—C11	120.70 (15)	N2—C29—C28	123.00 (15)
C12—C11—C10	120.39 (14)	N2—C29—H29	118.5
C16—C11—C10	120.58 (14)	C28—C29—H29	118.5
C16—C11—C12	119.03 (15)	O6—C30—N4	123.13 (16)
C11—C12—H12	119.7	O6—C30—C28	119.41 (15)
C13—C12—C11	120.53 (15)	N4—C30—C28	117.46 (15)
			
O3—Zn1—O1—C1	51.21 (14)	C4—C5—C6—C7	−2.2 (2)
N1—Zn1—O1—C1	−79.56 (11)	C8—C5—C6—C7	176.85 (16)
N2—Zn1—O1—C1	172.17 (11)	C4—C5—C8—C9	−63.8 (3)
O1—Zn1—O3—C10	44.51 (14)	C6—C5—C8—C9	117.2 (2)
N1—Zn1—O3—C10	177.80 (11)	C5—C6—C7—C2	−0.5 (2)
N2—Zn1—O3—C10	−72.79 (11)	O4—C10—C11—C16	−2.6 (2)
O1—Zn1—N1—C19	−39.06 (14)	O3—C10—C11—C16	178.05 (15)
O1—Zn1—N1—C23	146.03 (11)	O4—C10—C11—C12	177.73 (15)
O3—Zn1—N1—C19	170.13 (12)	O3—C10—C11—C12	−1.6 (2)
O3—Zn1—N1—C23	−4.78 (13)	C10—C11—C12—C13	179.96 (15)
N2—Zn1—N1—C19	60.87 (13)	C16—C11—C12—C13	0.3 (2)
N2—Zn1—N1—C23	−114.03 (12)	C10—C11—C16—C15	−178.82 (16)
O1—Zn1—N2—C25	−144.56 (13)	C12—C11—C16—C15	0.8 (3)
O1—Zn1—N2—C29	28.65 (12)	C14—C13—C12—C11	−1.3 (3)
O3—Zn1—N2—C25	0.90 (13)	C15—C14—C13—C12	1.0 (3)
O3—Zn1—N2—C29	174.12 (11)	C17—C14—C13—C12	−176.88 (16)
N1—Zn1—N2—C25	105.14 (13)	C13—C14—C17—C18	−60.5 (2)
N1—Zn1—N2—C29	−81.64 (12)	C15—C14—C17—C18	121.68 (19)
Zn1—O1—C1—O2	−4.6 (2)	C16—C15—C14—C13	0.1 (3)
Zn1—O1—C1—C2	175.06 (10)	C16—C15—C14—C17	178.02 (16)
Zn1—O3—C10—O4	−4.28 (19)	C11—C16—C15—C14	−1.1 (3)
Zn1—O3—C10—C11	175.04 (11)	N1—C19—C20—C21	−1.1 (2)
Zn1—N1—C19—C20	−174.76 (12)	C19—C20—C21—C22	0.9 (2)
C23—N1—C19—C20	0.2 (2)	C20—C21—C22—C23	0.2 (2)
Zn1—N1—C23—C22	176.15 (12)	C20—C21—C22—C24	177.08 (15)
C19—N1—C23—C22	1.0 (2)	C21—C22—C23—N1	−1.2 (2)
Zn1—N2—C25—C26	171.57 (13)	C24—C22—C23—N1	−178.25 (14)
C29—N2—C25—C26	−1.7 (2)	C21—C22—C24—O5	−136.93 (17)
Zn1—N2—C29—C28	−172.75 (12)	C21—C22—C24—N3	43.3 (2)
C25—N2—C29—C28	0.7 (2)	C23—C22—C24—O5	40.0 (2)
O1—C1—C2—C3	172.72 (14)	C23—C22—C24—N3	−139.73 (16)
O1—C1—C2—C7	−10.4 (2)	N2—C25—C26—C27	1.0 (3)
O2—C1—C2—C3	−7.6 (2)	C25—C26—C27—C28	0.8 (3)
O2—C1—C2—C7	169.20 (15)	C26—C27—C28—C29	−1.7 (2)
C1—C2—C3—C4	175.97 (15)	C26—C27—C28—C30	177.07 (15)
C7—C2—C3—C4	−0.9 (2)	C27—C28—C29—N2	1.0 (2)
C1—C2—C7—C6	−174.81 (15)	C30—C28—C29—N2	−177.71 (15)
C3—C2—C7—C6	2.1 (2)	C27—C28—C30—O6	8.4 (2)
C2—C3—C4—C5	−1.9 (3)	C27—C28—C30—N4	−171.28 (15)
C3—C4—C5—C6	3.4 (3)	C29—C28—C30—O6	−172.96 (16)
C3—C4—C5—C8	−175.67 (16)	C29—C28—C30—N4	7.4 (2)

### Hydrogen-bond geometry (Å, °)

**Table:**

*D*—H···*A*	*D*—H	H···*A*	*D*···*A*	*D*—H···*A*
N3—H32···O4^i^	0.86 (2)	1.99 (2)	2.833 (2)	165 (2)
N4—H41···O5^ii^	0.89 (2)	2.07 (2)	2.947 (2)	169 (2)
N4—H42···O6^iii^	0.84 (2)	2.06 (2)	2.901 (2)	177 (2)
C6—H6···O2^ii^	0.95	2.59	3.412 (2)	145
C19—H19···O5^ii^	0.95	2.29	3.2277 (19)	168
C21—H21···O2^i^	0.95	2.58	3.497 (2)	161
C23—H23···O3	0.95	2.49	3.085 (2)	121
C29—H29···O5^ii^	0.95	2.45	3.299 (2)	149
C17—H17A···Cg1^iv^	0.99	2.63	3.436 (2)	139
C20—H20···Cg1^v^	0.95	2.77	3.603 (2)	147

Symmetry codes: (i) −*x*+1, *y*+1/2, −*z*+3/2; (ii) *x*+1, *y*, *z*; (iii) −*x*+3, −*y*+2, −*z*+2; (iv) −*x*+1, *y*+1/2, −*z*+1/2; (v) −*x*, *y*−1/2, −*z*+1/2.
